# Supplementary figures and images for: A new tandem repeat-based genotyping scheme for the global surveillance of Xanthomonas citri pv. mangiferaeindicae, an understudied bacterial pathogen of major importance to mango and cashew production
Source: PLoS One. 2025 Nov 26;20(11):e0336768. doi: 10.1371/journal.pone.0336768 (PMC12654951; doi:10.1371/journal.pone.0336768)

**Fig. S1**

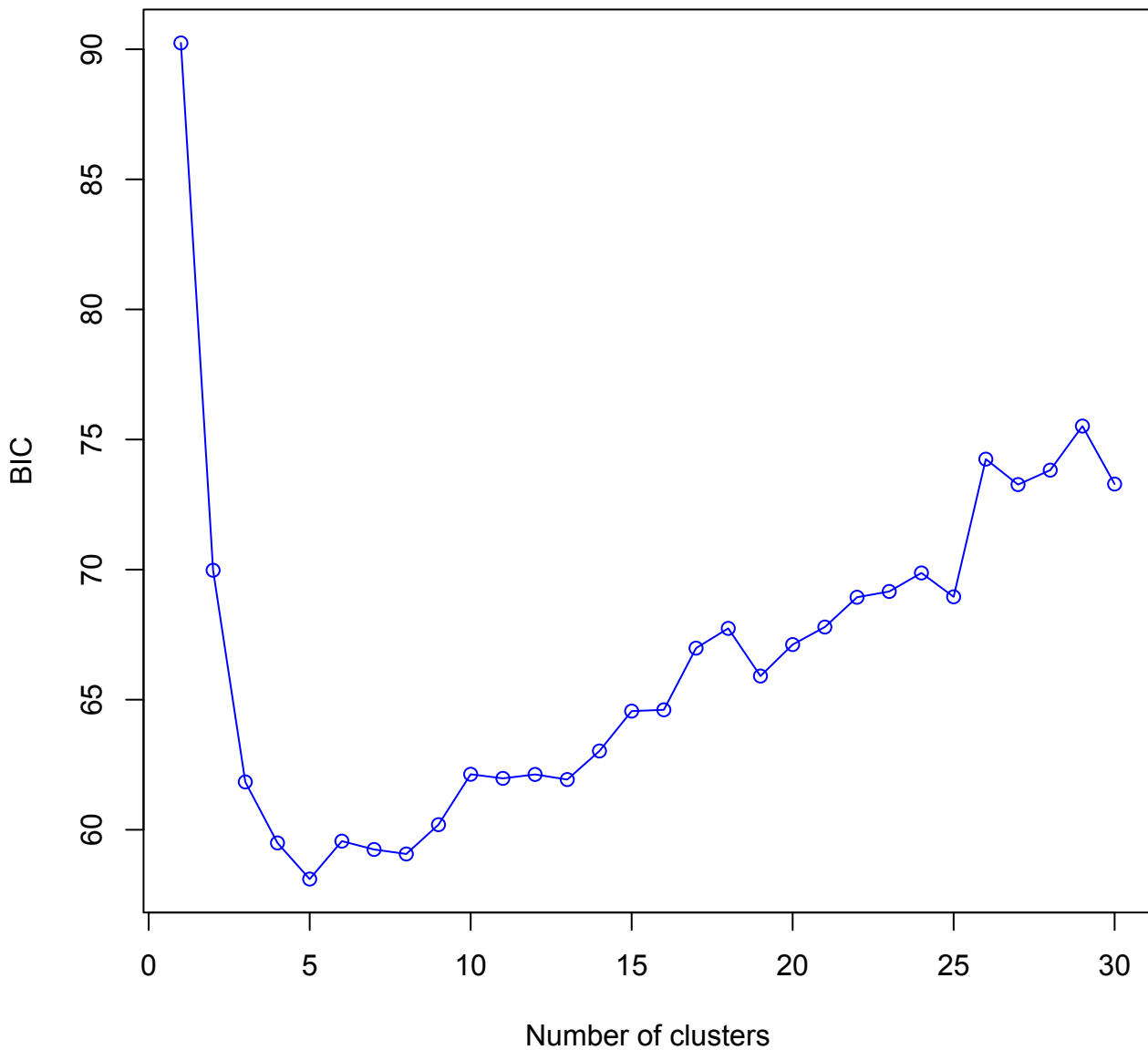

Supplement: S1 Fig — (PDF) [file pone.0336768.s001.pdf]
