# Supplementary material for: A new tandem repeat-based genotyping scheme for the global surveillance of Xanthomonas citri pv. mangiferaeindicae, an understudied bacterial pathogen of major importance to mango and cashew production
Source: PLoS One. 2025 Nov 26;20(11):e0336768. doi: 10.1371/journal.pone.0336768 (PMC12654951; doi:10.1371/journal.pone.0336768)
Supplement: Attachment — Submitted filename: reviews.docx [file pone.0336768.s006.docx]

Reviewer #1: Major comments：

Xanthomonas citri pv. mangiferaeindicae (Xcm) is the causal agent of bacterial canker, also named bacterial black spot or angular spot, in some species of Anacardiaceae family. With the worldwide introduction and expanded cultivation of certain Anacardiaceae plants with significant economic value, bacterial canker disease has also been continuously spreading and worsening. However, compared to many other xanthomonad pathogens, Xcm has not received sufficient attention. Molecular typing is the basis for population genetics and tracing of the pathogens. The genetic structure of Xcm was primarily dissected by amplified fragment length polymorphism (AFLP), an old typing method with randomness and lacking of sequence information. The development of genomics has provided some new typing methods with more precise, faster, higher in resolution, and easier to operate. In this study, Boyer and her colleagues developed a new tandem repeat-based genotyping scheme targeting 16 TR markers in Xcm, and the MLVA-16 dataset was deposited in a dedicated online database. The MLVA-16 genotyping scheme was only slightly less discriminative than AFLP. Data derived from the two datasets were highly correlated, suggesting a fairly good resolution and phylogenetic signal. This study will further expand our understanding of the genetic diversity of Xcm, and will be helpful for disease diagnosis, early warning, and quarantine of this neglected bacterial pathogen with agricultural risks. The manuscript is well designed and written, and the content is suitable for publication in PLoS ONE. However, there are still some parts that are worth polishing and improving.

We thank reviewer#1 for his/her kind comments.

Questions and suggestions:

1.Tandem repeats are found throughout the genomes, in which the number of repeat units can vary due to slipped-strand mispairing during DNA replication, leading to length polymorphisms. TR typing is manifesting as one kind of genetic fingerprinting of individual organism, indicating that the individual has specific TR markers. It is suggested to construct a pan-MLVA marker library (MLVAbank database) using several representative and high-quality sequenced Xcm strains other than one single Xcm strain. This additional task will not affect the original MLVA-16 genotyping scheme in the manuscript.

Indeed, this was partly achieved in the original submission, as MLVA data from the high-quality genome sequences were already included in the MLVAbank online database. We did not construct a new database in MLVAbank, as suggested, as we believe that it is of limited interest to the readership. Instead, we include in the revised version a table summarizing the total minisatellite content of each sequenced strain for reviewers’ appreciation (including the loci that were not selected for MLVA-16 typing), as determined by Tandem Repeat Finder and the filtering criteria we used. If the editor feels that it is of sufficient interest, it may be included as a supplementary table in the published version of our manuscript.

2.The manuscript is about the establishing an experimental method or scheme? which needs to have a clear logic and a well-defined technical route. It is recommended to add a technical route map or a research scheme map in the first part of Results.

In our opinion, the input of the present study is more providing a new genotyping scheme rather than providing an experimental method, as our analytical procedure was really standard, and is highly similar to the one used previously in many molecular epidemiological studies of human, animal or plant bacterial pathogens. Nevertheless, we followed reviewer #1’s recommendation and included at the beginning of the Results section a short paragraph summarizing our technical route.

3.There are only one table and two figures in the main text of this manuscript, seeming somewhat thin and lacking in information. It is better to add a genomic collinear diagram of several representative strains, along with each TR marker in certain genome.

A new Fig. 1 was produced as requested.

4.Please also pay attentions to the typo errors, including misplaced space and punctuation marks, e.g., Line 30, misplaced space; Line 279, [24]]; Line 148, 2X, 2×; Line 149, 5X, 5×; Line 170, 10X, 10×.

Modified as requested.

Reviewer #2: Thank you for submitting an interesting and useful study. This research highlights the relevance of using MVLA as a tool to study the population diversity of bacterial pathogens.

I have some really minor comments:

lines 69 and 70 Vietnam not Viet Nam

line 78 should be Gram not gram as it is named affer a person

Line 79 incomplete sentence, unlike most Xanthomonas species?

Line 297 what Rhus species?

All editorial suggestions placed by reviewer #2 were included in the revised version. The vernacular name of *Searsia longipes* remains the large-leaved rhus, although its genus name was modified from *Rhus* to *Searsia* in 2007 and further validated in Catalog of Life.
